# 

**Published:** 1915-07

**Authors:** 


### INDEX TO VOLUME XXXI.

### ORIGINAL ARTICLES.

Page

Carcinoma of the Colon: Its Pathology and Symptoms. By Claude C. Cody, M. A., M. D. .... 1

The X-ray in the Treatment of Malignancy. By B. T. Van Zandt, M. D., Houston, Texas .... 8

The Eradication of Tuberculosis and the Means by Which it May Be Accomplished. By F. C. Smith, M. D., U. S. Public Health Service .... 43

Diarrhea Diseases in Infancy. By Dr. A. Spencer Davis, Dallas, Texas .... 50

A Few Surgical Don'ts. By Chas. H. Venable, M. D. .... 53

Skiagraphic Study of Thorax, Thoracic Wall and Thoracic Viscera. By L. B. Bibb, M. D., and Gilliland, M. D., Austin, Texas .... 87

Piles or Hemorrhoids. By Dr. James A. Hill, Houston, Texas .... 131

President's Address—The Texas Surgical Society. By James E. Thompson, M. D., Galveston, Texas .... 171

The Thyroid Gland. By W. L. Crosthwait, M. D., Waco, Texas .... 183

Hypo and Hyperthyroidism. By J. S. Hixson, M. D., San Angelo, Texas .... 223

Dislocation of Elbow Joint and Flexed Ankylosis of Knee Joint. By Joe Gilbert, M. D., F. A. C. S., Austin, Texas .... 226

Symptoms of Chronic Appendicitis. By Wade H. Walker, M. D., Wichita Falls, Texas .... 229

Recurrent Bronchitis. By Dr. L. P. Tenny, Troup, Texas .... 231

The Mystery of Infection. By Dr. G. Henri Bogart, Shelbyville, Ill .... 234

Hemoptysis—Report of Three Cases Treated by Artificial Pneumothorax. By Alvis E. Greer, M. D., Houston, Texas .... 265

The New Discovery About Pneumonia. By Dr. Leonard Keen Hirshberg, A. B., M. A., M. D., Johns Hopkins .... 269

Involution Melancholia. By J. A. McIntosh, M. D., San Antonio, Texas .... 307

Diphtheria. By M. L. Wilbanks, M. D., Greenville, Texas .... 312

Diagnosis of Syphilis by Laboratory Methods. By Henry H. Morton, M. D., Clinical Professor of Genito-Urinary Diseases in the Long Island Hospital; Genito-Urinary Surgeon to Long Island College and Kings County Hospital and the Polhemus Memorial Clinic, etc., Brooklyn, New York .... 318

How Will the War Now Raging Be of Benefit to Mankind? By F. Paschal, M. D., F. A. C. S., San Antonio, Texas .... 351

Surgical Treatment of Retro-Displacements of the Uterus. By Dr. James A. Hill, Houston, Texas .... 354

A Contribution to the Study of Hernias of the Ovary, of the Fallopian Tube, and of the Ovary and Fallopian Tube. By Aime Paul Heineck, M. D., Chicago, Ill., Surgeon to the Francis Willard, Jefferson Park and Rhodes Avenue Hospitals, etc. .... 356

431959

Page
Are Dents in the Forehead Inherited? By Dr. Leonard Keen Hirshberg, A. B., M. A., M. D. (Johns Hopkins) ............ 365
Vincent's Angina. By Dr. Ray Karchmer Daily, Houston, Texas. 393
Difficulties in Breast Feeding. Mary Cleveland Harper, M. D., San Antonio, Texas ........................................ 397
Clean Milk. By Ethel Lyon Heard, Galveston, Texas .......... 406
An Unusual Case of Renal Tuberculosis. Violet H. Keiller, A. B., M. D., Galveston, Texas ................................ 409
Abnormal Delinquency. Minnie L. Maffett, M. D., Dallas, Texas. 411
The Examination of the Blood Smear as a Diagnostic Asset. Martha A. Wood, M. D., Houston, Texas ................... 420
The Treatment of Neuralgia by Alcoholic and Other Injections. By G. Wilse Robinson, M. D., Kansas City, Missouri .......... 441
The Diagnosis of the Internal Secretory Disorders. By Henry R. Harrower, M. D., F. R. S. M. (Lond.), Los Angeles, Cal...... 449
Malignant Disease of the Skin. By Dr. W. L. Crosthwait, Waco, Texas ............................................................ 454
Old-Fashioned Drugs. By Dr. G. Henri Bogart, Shelbyville, Ill.. 460
Just Some Observations on Childhood. By E. S. Goodhue, A. M., M. D., LL. D., Hawaii ........................................ 462
The Diagnosis of Internal Secretory Disorders. (Article No. 2.) By Dr. Henry R. Harrower, M. D., F. R. S. M., Los Angeles, California ........................................................ 487
Vaccines in Mixed Pulmonary Infection. By Theo Y. Hull, M. D., San Antonio, Texas ......................................... 491
Congenital Pyloric Stenosis. By Joe Gilbert, M. D., Austin, Texas ............................................................ 499
Some Observations on Childhood. By E. S. Goodhue, A. M., M. D., LL. D. ....................................................... 503
Twilight Sleep. By Dr. J. B. Dorrell, Springfield, Mo......... 509

### DEPARTMENT OF EUGENICS.

(Conducted by Dr. Malone Duggan, San Antonio, Texas.)

Heredity and Cancer. By Dr. Malone Duggan ............... 12
A Warning. By Dr. Malone Duggan ......................... 12
Higher Education and Race Suicide. By Dr. Malone Duggan... 13
The Evolution of Character. By Dr. Malone Duggan .......... 14
Legal Limitation of Family Limitation. By Dr. G. Henri Bogart, Paris, Ill .............................................. 17
Is War Dysgenic? Exchange .................................. 55
The Rôle of the Farmer as an Eugenist. By Dr. Malone Duggan. 56
War Babies and the Mother. By Dr. Malone Duggan .......... 58
A Significant Meeting. By Dr. Malone Duggan ............... 59
Rural Morality. By Dr. Malone Duggan ...................... 61
The Doctor Himself. Exchange ............................... 63
Dr. Kellog on Aristocracy .................................. 107
Heredity and Environment. By Dr. Malone Duggan ........... 108
Genetic Philosophy. By Dr. Malone Duggan .................. 137
Eugenics of War. Exchange ................................. 140

Page
The Inheritance of Temperament. By Dr. Malone Duggan..... 144
Triumphant Motherhood. By Dr. G. Henri Bogart............ 145
Burbank on Race Betterment. By Dr. Malone Duggan......... 146
Alcohol, Syphilis, and Gonorrhea, on Death Certificates. Exchange ........................................... 191
Genetic Sentilations. By Dr. Malone Duggan................ 192
To The Rescue. By Dr. Malone Duggan...................... 195
The Moral Transition. By Dr. Malone Duggan................ 196
Infant Mortality and Natural Selection. By Dr. Malone Duggan. 236
The Effects of Selection. By Dr. Malone Duggan............. 236
Biology to the Fore Again. By Dr. Malone Duggan........... 237
Short Cut to Eugenics. By Dr. Malone Duggan.............. 238
Sane at Last. By Dr. Malone Duggan....................... 240
Mental Efficiency. By Dr. Malone Duggan.................... 273
Eugenic and Euthenic Notes. By Luther Burbank, Geo. E. Johnson, Dr. W. H. B. Aikens, Irving Fisher, President Hyde...... 275
Value of Negative Eugenics. By Edwin G. Conklin, Professor of Biology, Princeton University, Princeton, N. J............ 276
Extracts from an Address Delivered in Chicago, December 10, 1915, before the Eugenics Educational Society. By Casper L. Redfield ........................................... 321
Is Contraception Injurious to Health? By C. V. Drysdale, B. Sc., in the Malthusian, December 15th...................... 324

### EDITORIAL DEPARTMENT.

The Waste of Life from Cancer. By John T. Moore, A. M., M. D., Fellow American College of Surgeons, Houston, Texas.... 23
Articles of Faith Concerning Cancer—A Platform Upon Which to Unite in the Campaign of Education.......................... 29
Words of Encouragement............................31, 32, 33
Medical Revolutions. By T. D. Crothers, M. D., Hartford, Conn. 64
Justifiable Scope of Medical Journal Advertising. By Dr. G. Henri Bogart, Paris, Ill.................................. 66
Pellagra. By Dr. R. H. L. Bibb............................ 68
The Family Physician. By T. D. Crothers, M. D., Hartford, Conn. ................................................ 113
War Babies ............................................... 116
Medical Ethics. By Dr. J. H. Florence, Houston, Texas....... 148
Physicians Should be Students............................ 153
Editorial Comment ....................................156-159
Fads in Medicine. By Dr. G. Henri Bogart, Shelbyville, Ill..... 200
Meeting of Southern Medical Association.................... 201
Medical History of Texas. By Hugh W. Crouse.............. 204
Two Great Men Gone...................................... 205
Medical Editors' Association.............................. 243
Public Health Service Discovers Cause and Cure of Pellagra—Pellagra Caused by Insufficient Proteid Diet................ 245
The American Society for the Study of Alcohol and Other Narcotics ................................................ 247

INDEX.

Page

Seize Substitute Specifics.......................................... 248

Report of Clinic on Pellagra Held in Dallas, Texas, in Connection with Meeting of Southern Medical Association, November 8-11, 1915. By John R. Lehmann, A. B., M. D., Dallas, Texas. 249

Scintillations from the Pellagra Symposium.................. 253

Dallas Clinics .................................................... 255

The New Year..................................................... 282

Typhoid Responsibility. By Dr. G. Henri Bogart, Shelbyville, Ill. 286

The Doctor—Clipping ........................................... 330

Exterminate the Pests.......................................... 331

Safeguard Ethics ................................................ 334

Rats and Mice.................................................... 334

It Isn't Your Town—It's You. Clipped..................... 367

An Important Announcement................................. 367

What Organization May Accomplish........................ 368

Average Pay is Poor............................................. 370

Leprosy is Spreading........................................... 370

Advances in Surgery............................................ 370

Medical Inspection ............................................. 371

Cities Without Flies............................................ 371

Collections and Common Sense............................. 372

To Subscribers ................................................... 425

Our Forthcoming Series of Articles......................... 425

This Number of the Journal.................................. 426

This is Your Journal............................................ 427

Dr. White's Articles............................................ 428

The Cost of Disease............................................ 468

They Show Progress............................................ 468

Fighting Diseases ............................................... 469

Pellagra Preventable ........................................... 469

County Hospitals ................................................ 470

A Valuable Series................................................ 470

This Journal is Yours........................................... 470

The Physician's Opportunity................................. 513

Some Things for the Future................................... 513

Abstracts and Selections.....71, 72, 76, 159, 162, 163, 164, 165, 471

Items of General Interest........................................
..33, 77, 119, 208, 258, 287, 291, 292, 293, 335, 374, 428, 474, 518

Helpful Hints for the Busy Doctor............................
..........40, 84, 128, 174, 220, 262, 302, 347, 390, 438, 482, 526

Book Reviews.........................38, 82, 172, 299, 344, 436, 480

### Deaths.

Dr. G. W. Gotcher, of Garza, died at his home May 20, 1915.

Dr. R. N. Younger died in Marlin May 17, 1915.

Dr. J. P. Dickerson died at Tazewell May 8, 1915.

Dr. Guy Minter died at his home, Pine Forest, May 21, 1915.

Dr. Frank Douglas died May 17th at his home in Covington.

Dr. A. M. Anderson, formerly of Eden, died at his home in Throckmorton.

Dr. John Hampton, of Waxahachie, died in a sanitarium in Chicago June 5, 1915.

Dr. J. H. Yarbrough died in Dallas May 24th. His remains were sent to Stamps, Ark.

Dr. Geo. P. Hall, of Houston, regarded as one of the leading physicians of the State, died at Houston on June 9, 1915.

Dr. J. H. Riley, of Denton, aged 101, died at his home on the 12th of June.

Dr. R. F. White, a resident of Texas for fifty years, died at his home, Prairie Hill, May 22, 1915.

